# Efficient Secretory Production of Lytic Polysaccharide Monooxygenase *Ba*LPMO10 and Its Application in Plant Biomass Conversion

**DOI:** 10.3390/ijms24119710

**Published:** 2023-06-03

**Authors:** Xiao Guo, Yajing An, Fuping Lu, Fufeng Liu, Bo Wang

**Affiliations:** 1 College of Chemical and Biological Engineering, Shandong University of Science and Technology, Qingdao 266590, China; 2 Key Laboratory of Industrial Fermentation Microbiology of the Ministry of Education, College of Biotechnology, Tianjin University of Science and Technology, Tianjin 300450, China

**Keywords:** lytic polysaccharide monooxygenase, fermentation, enzyme properties, biomass conversion, reducing sugar, surface morphology

## Abstract

Lytic polysaccharide monooxygenases (LPMOs) can oxidatively break the glycosidic bonds of crystalline cellulose, providing more actionable sites for cellulase to facilitate the conversion of cellulose to cello-oligosaccharides, cellobiose and glucose. In this work, a bioinformatics analysis of *Ba*LPMO10 revealed that it is a hydrophobic, stable and secreted protein. By optimizing the fermentation conditions, the highest protein secretion level was found at a IPTG concentration of 0.5 mM and 20 h of fermentation at 37 °C, with a yield of 20 mg/L and purity > 95%. The effect of metal ions on the enzyme activity of *Ba*LPMO10 was measured, and it was found that 10 mM Ca^2+^ and Na^+^ increased the enzyme activity by 47.8% and 98.0%, respectively. However, DTT, EDTA and five organic reagents inhibited the enzyme activity of *Ba*LPMO10. Finally, *Ba*LPMO10 was applied in biomass conversion. The degradation of corn stover pretreated with different steam explosions was performed. *Ba*LPMO10 and cellulase had the best synergistic degradation effect on corn stover pretreated at 200 °C for 12 min, improving reducing sugars by 9.2% compared to cellulase alone. *Ba*LPMO10 was found to be the most efficient for ethylenediamine-pretreated Caragana korshinskii by degrading three different biomasses, increasing the content of reducing sugars by 40.5% compared to cellulase alone following co-degradation with cellulase for 48 h. The results of scanning electron microscopy revealed that *Ba*LPMO10 disrupted the structure of Caragana korshinskii, making its surface coarse and poriferous, which increased the accessibility of other enzymes and thus promoted the process of conversion. These findings provide guidance for improving the efficiency of enzymatic digestion of lignocellulosic biomass.

## 1. Introduction

Cello-oligosaccharide, cellobiose and glucose are the main degradation products of cellulose. Cello-oligosaccharide is a functional oligosaccharide that can strengthen human immunity to prevent the occurrence of diseases [1,2], and can also change the bacterial composition of animal intestines to promote intestinal development [3]. Because of its low sweetness properties, cellobiose is often used as a food additive in the beverage industry [4,5]. It is a sweetener very friendly to people who suffer from diabetes. In addition, glucose, the smallest unit of cellulose, can alleviate the energy shortage by converting it into bioethanol through fermentation [6].

Among the methods for the preparation of cello-oligosaccharide, cellobiose and glucose, enzymatic techniques are widely used because of their low cost, mild conditions and environmental friendliness. The cellulose structure is complex and mostly crystalline, while the current cellulases have low catalytic efficiency for crystalline cellulose, which limits the conversion efficiency of cellulose [7,8]. Recently, a class of copper ion-dependent lytic polysaccharide monooxygenases (LPMOs) was identified which can disrupt crystalline polysaccharides and improve substrate accessibility [9,10]. In the presence of an electron donor, the divalent copper ion in the active center of the LPMO receives an electron, thus becoming a monovalent copper ion, which in turn breaks the glycosidic bond of the polysaccharide [11,12]. Therefore, the synergistic effect of LPMO and cellulase can significantly improve the conversion efficiency of cellulose [10,13,14,15].

Based on amino acid sequences and functional similarities, LPMOs are currently classified into eight auxiliary activities (AA) families, including AA9–AA11 [16,17] and AA13-AA17 [18,19,20,21,22]. Among them, AA9 and AA10 are the two earliest discovered and most studied families [17,23,24]. In our previous study, a new AA10 protein (*Ba*LPMO10) was obtained by screening and characterization, and showed catalytic activity for both cellulose and chitin, which is important for the conversion of biomass [25]. *Ba*LPMO10 was expressed extracellularly in *Escherichia coli* (*E. coli*) using signal peptides, but in low yields [25]. The fermentation process is critical to the level of protein secretion, so optimizing the fermentation conditions can improve the level of protein secretion.

In order to further investigate the biochemical properties of *Ba*LPMO10, in this study, we analyzed its bioinformatics and then improved its production by optimizing the fermentation conditions, including isopropyl β-D-1-thiogalactopyranoside (IPTG) concentration, fermentation temperature and time. In addition, the effects of metal ions, reducing agent DTT, chelating agent EDTA and organic reagents on the enzymatic activity and kinetic parameters of *Ba*LPMO10 were determined. Finally, the degradation ability of *Ba*LPMO10 for pretreated corn stover, rice straw, Caragana korshinskii and pulp was analyzed, and the surface structure of the biomass was observed in order to determine the mode of action of *Ba*LPMO10.

## 2. Results and Discussion

### 2.1. Bioinformatics Analysis of BaLPMO10

To explore the bioinformatics of *Ba*LPMO10, its physicochemical properties were analyzed using the ProtParam website (https://web.expasy.org/protparam/) (accessed on 10 March 2021) [26]. The results indicated that it was composed of 206 amino acids, with a molecular formula of C_1017_H_1534_N_272_O_301_S_2_ and a theoretical molecular weight of 22451.13 Da. The isoelectric point was determined to be 8.62, with 20 positively charged amino acid residues (R + K) and 18 negatively charged amino acid residues (D + E). *Ba*LPMO10 had a total average hydrophilicity of −0.388 (<0), indicating that it was a hydrophilic protein. The instability index was calculated to be 30.72 (<40), suggesting that *Ba*LPMO10 was a stable protein. Additionally, the TMHMM Server 2.0 website (http://www.cbs.dtu.dk/services/TMHMM-2.0/) (accessed on 10 March 2021) [27] was used to predict whether *Ba*LPMO10 contained any transmembrane regions, and the results shown in Figure S1 indicate that *Ba*LPMO10 did not have any transmembrane domains. To predict whether *Ba*LPMO10 contained a signal peptide, its amino acid sequence was analyzed using the SignalP 5.0 Server (http://www.cbs.dtu.dk/services/SignalP-5.0/) (accessed on 10 March 2021) [28]. The results, as shown in Figure S2, revealed that *Ba*LPMO10 contained a 27-amino-acid signal peptide (MKGLVKAAVLTVTLGIGGAFYSSDASA), indicating that it was a secretory protein.

### 2.2. Optimization of BaLPMO10 Fermentation Conditions

In the previous study, the gene of *Ba*LPMO10 was successfully constructed into the vector pET-22b and achieved secretory expression using its native signal peptide [25]. In order to improve the secretion level of *Ba*LPMO10 and reduce the cost of industrial downstream processing, its fermentation conditions were optimized.

#### 2.2.1. Optimization of IPTG Induction Concentration

**Figure 1:**
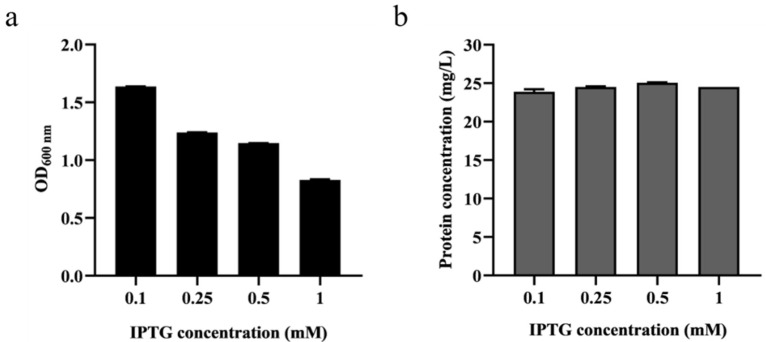
Effect of various IPTG concentrations on cell growth and *Ba*LPMO10 expression. (**a**) Cell concentration; (**b**) Production of *Ba*LPMO10.

Due to the fact that pET-22b operates through an inducible T7 promoter, the concentration of the IPTG inducer plays a critical role in the expression of the target protein. Therefore, to obtain the appropriate IPTG concentration, it was added to final concentrations of 0.1, 0.25, 0.5 and 1 mM for induction during the fermentation process of *Ba*LPMO10. Following fermentation, we measured the cell density at OD_600_ (Figure 1a) and collected the purified protein from the supernatant to determine its concentration (Figure 1b). Figure 1a demonstrates that, as the IPTG concentration increased, the cell concentration at OD_600_ decreased, suggesting that IPTG had a toxic effect that inhibited bacterial growth [29]. Figure 1b reveals that the secretion level of the *Ba*LPMO10 was highest when the IPTG induction concentration was 0.5 mM, indicating that this was the optimal concentration for IPTG induction. However, the concentration of *Ba*LPMO10 was reduced in the system with the addition of 1 mM IPTG. Higher concentrations of IPTG may adversely affect bacterial growth and thus interfere with the expression and secretion levels of the target protein. Similar results were found in previous studies [30], suggesting that an IPTG concentration of 0.5 mM may be the most appropriate when using *E. coli* to express exogenous proteins.

#### 2.2.2. Optimization of Fermentation Temperature and Time

**Figure 2:**
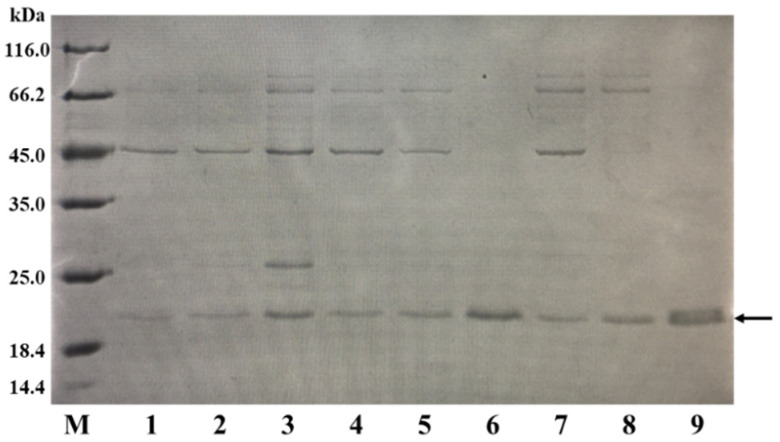
Comparison of the *Ba*LPMO10 production at various temperatures and times. The band indicated by the arrow is *Ba*LPMO10. 1: 16 °C, 4 h; 2: 16 °C, 8 h; 3: 16 °C, 20 h; 4: 30 °C, 4 h; 5: 30 °C, 8 h; 6: 30 °C, 20 h; 7: 37 °C, 4 h; 8: 37 °C, 8 h; 9: 37 °C, 20 h.

When expressing different exogenous proteins in a host, the optimal induction temperature and time can vary. To determine the ideal temperature and time for the expression of *Ba*LPMO10, we investigated the effects of various fermentation temperatures and times on the secretory expression of *Ba*LPMO10. After the addition of an IPTG inducer to the fermentation medium, the fermentation was carried out at 16 °C, 30 °C and 37 °C for 4 h, 8 h and 20 h, respectively. *Ba*LPMO10 in the supernatant was purified at the end of fermentation, and the protein purity was analyzed by SDS-PAGE under different fermentation conditions. The results are shown in Figure 2. The results demonstrated that the highest expression level of *Ba*LPMO10, with the least amount of impurities, was obtained after 20 h of fermentation at 37 °C (lane 9). The protein yield from purifying 1 L of fermentation broth was 20 mg. These findings highlight the critical role of IPTG concentration, fermentation temperature and time in regulating the expression of target proteins, and underscore the importance of careful optimization for maximizing protein expression.

### 2.3. Enzymatic Properties of BaLPMO10

**Figure 3:**
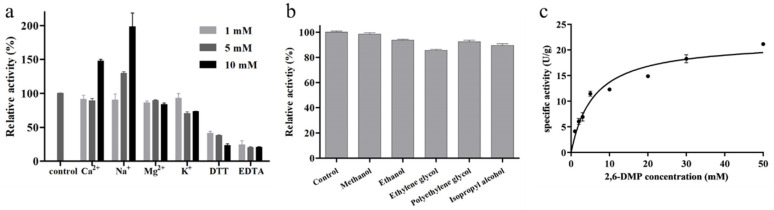
Enzymatic properties of *Ba*LPMO10. (**a**) Effect of metal ions, DTT and EDTA on the activity of *Ba*LPMO10. (**b**) Effect of organic reagents on the activity of *Ba*LPMO10. (**c**) Kinetic plot of *Ba*LPMO10.

In previous studies, we investigated the effects of temperature and pH on the activity of *Ba*LPMO10 and found that its optimal temperature was 70 °C, its optimal pH was 6.0 and its maximum specific activity was 91.4 U/g [25]. As *Ba*LPMO10 was a metalloenzyme, the effects of different metal ions (K^+^, Na^+^, Ca^2+^, Ba^2+^, Mg^2+^, Cu^2+^, Mn^2+^, Co^2+^ and Fe^3+^), DTT and EDTA on its activity were examined. However, when Ba^2+^, Cu^2+^, Mn^2+^, Co^2+^ and Fe^3+^ were added, the reaction mixture became turbid, so these five metal ions were not studied further. The effects of the other four metal ions on *Ba*LPMO10 activity are shown in Figure 3a. High concentrations of Ca^2+^ promoted enzyme activity; 10 mM Ca^2+^ increased *Ba*LPMO10 activity by 47.8%, and 5 mM and 10 mM Na^+^ increased *Ba*LPMO10 activity by 29.5% and 98.0%, respectively. However, different concentrations of Mg^2+^ and K^+^ inhibited enzyme activity. By comparison with other LPMOs, it was found that different metal ions showed inconsistent results regarding their enzyme activity. For example, different concentrations of Mg^2+^ were able to increase the enzyme activity of *Mt*C1LPMO; 10 mM of Mg^2+^ increased the enzyme activity of *Mt*C1LPMO by 37.6% [31]. In addition, the reducing agent DTT and chelating agent EDTA both inhibited enzyme activity in a concentration-dependent manner (Figure 3a). DTT and EDTA at 10 mM inhibited the enzyme activity of *Ba*LPMO10 by 76.9% and 79.6%, respectively. Both of these are commonly used protease inhibitors. EDTA is supposed to chelate the copper ions in the active center of *Ba*LPMO10, resulting in a more pronounced decrease in enzyme activity.

The impacts of five organic reagents, namely, methanol, ethanol, ethylene glycol, polyethylene glycol and isopropyl alcohol, on the enzyme activity of *Ba*LPMO10 were analyzed, and the results are presented in Figure 3b. It can be seen that all five organic reagents reduced the enzyme activity to different degrees. Among them, ethylene glycol exhibited the greatest impact on enzyme activity, with a 14.5% decrease in *Ba*LPMO10 activity. Methanol had the lowest impact on *Ba*LPMO10 activity, with only a 1.7% decrease in activity. Ethanol, polyethylene glycol and isopropyl alcohol showed intermediate impacts on *Ba*LPMO10 activity, with the activity decreasing by 6.4%, 7.6% and 10.6%, respectively.

To clarify the enzyme kinetics of *Ba*LPMO10, its enzymatic activity was determined using different concentrations of 2,6-DMP as a substrate. The results of the kinetic curves are shown in Figure 3c. Based on the kinetic curves, the kinetic parameters of *Ba*LPMO10 were calculated, and the results are shown in Table 1. The maximum velocity (*V*_max_), the substrate affinity (*K*_m_) and the catalytic efficiency (*k*_cat_/*K*_m_) of *Ba*LPMO10 were 21.78 ± 2.23 U/g, 5.89 ± 1.95 mmol/L and 18.48 s^−1^ mM^−1^, respectively. The *K*_m_ of *Ba*LPMO10 was lower than that of *Nc*LPMO9C (245 ± 74 mmol/L) [32], indicating a higher affinity between *Ba*LPMO10 and the substrate.

### 2.4. Synergistic Degradation of Steam-Exploded Corn Stover by BaLPMO10 and Cellulase

**Figure 4:**
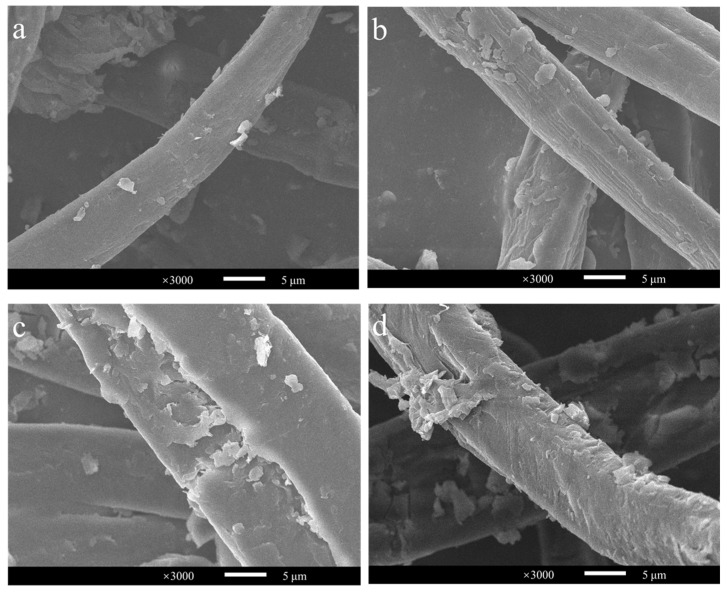
Observation of the morphology of steam-exploded corn stover. (**a**) 200 °C, 3 min; (**b**) 200 °C, 6 min; (**c**) 200 °C, 9 min; (**d**) 200 °C, 12 min.

Approximately hundreds of millions of tons of corn stover are wasted each year in China, and it has a very high potential for conversion into cello-oligosaccharides, cellobiose and glucose [33,34]. Its structure is complex, and pretreatment would greatly improve its bioavailability. Compared with acid and alkali pretreatment, steam explosion can reduce the use of organic chemicals, making it a green pretreatment method [35,36]. Therefore, corn stover with steam explosions at 200 °C for 3, 6, 9, and 12 min was chosen as the substrate with which to investigate the conversion efficiency of *Ba*LPMO10 on corn stover. The morphology of the cellulose in the four pretreated corn stover samples was observed using scanning electron microscopy (SEM) (Figure 4), and obvious differences in cellulose morphology were observed. The surface morphology of the cellulose in the sample pretreated for 3 min showed the smallest changes (Figure 4a), indicating the lowest degree of disruption. The sample which showed the most severe destruction was that pretreated for 12 min (Figure 4d), which was able to increase the accessibility of the enzyme. As the pretreatment time increased, the cellulose surface clearly continued to rupture. This is because the longer pretreatment time allowed more hot steam to penetrate into the internal voids of the fibers, enhancing the effect of the steam explosion pretreatment and further decomposing the fiber structure [35,37,38].

**Figure 5:**
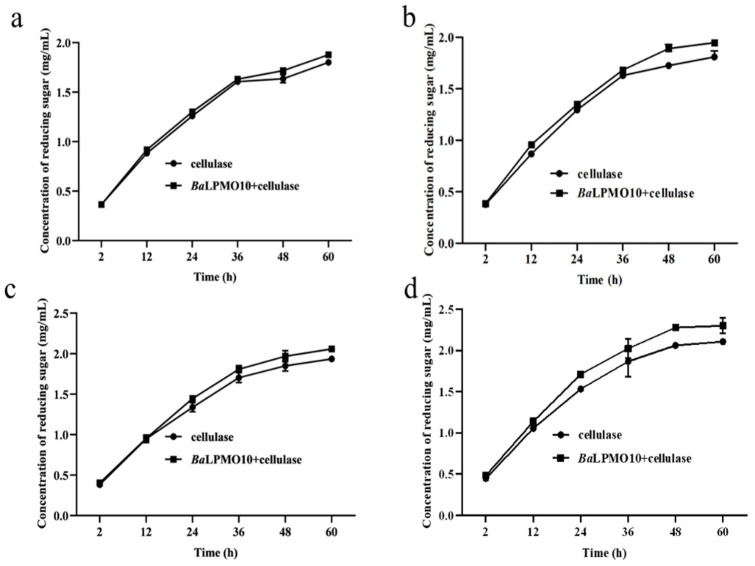
Synergism activity of *Ba*LPMO10 and cellulase on corn stover with different pretreatments. (**a**) 200 °C, 3 min; (**b**) 200 °C, 6 min; (**c**) 200 °C, 9 min; (**d**) 200 °C, 12 min.

In order to select the optimal pretreatment condition for *Ba*LPMO10, the synergistic degradation of corn stover with different pretreatments by *Ba*LPMO10 and cellulase was investigated, and the results are shown in Figure 5. It was found that the synergistic actions of *Ba*LPMO10 and cellulase on corn stover under different pretreatment conditions were different. As the pretreatment time increased, the reducing sugar content produced by enzymatic digestion increased. For the corn stover pretreated for 12 min, the highest reducing sugar concentration was produced after 60 h of degradation by either cellulase alone or the two enzymes working together, with concentrations of 2.1 mg/mL and 2.3 mg/mL, respectively (Figure 5d). However, the degree of reducing sugars enhanced by the synergistic action of the two enzymes did not increase along with the substrate pretreatment time. The reducing sugar content of corn stover pretreated for 3, 6, 9 and 12 min was increased by 4.2% (Figure 5a), 7.6% (Figure 5b), 6.3% (Figure 5c) and 9.2% (Figure 5d), respectively, after 60 h of synergistic degradation by the two enzymes rather than cellulase alone. The corn stover pretreated for 3 min released the lowest amount of reducing sugar (Figure 5a), possibly due to the low degree of structural damage caused by the short pretreatment time (Figure 4a), which made it difficult for the enzyme to contact the substrate. Surprisingly, the reducing sugar content raised by *Ba*LPMO10 in synergy with the cellulase degradation of corn stover pretreated for 6 min (7.6%) was higher than that with the 9 min treatment (6.3%), probably due to the incomplete pretreatment resulting in a high lignin content. Lignin can provide electrons to LPMO, thereby increasing its catalytic efficiency [13,15,39,40]. The reducing sugars released from the corn stover with pretreatment for12 min were the highest, which is likely due to the more thorough destruction of the lignocellulosic structure and the higher exposure of cellulose caused by the longer pretreatment time, which made it easier for the enzyme to contact the cellulose. However, the longer the pretreatment time, the higher the energy consumption. Because the pretreatment temperature was 200 °C, both equipment and material were required to be heated from room temperature to 200 °C until the end of the pretreatment, and the steam consumption was very high. Therefore, a comprehensive consideration of economic costs is also a future consideration.

### 2.5. Synergistic Degradation of Pretreated Rice Straw, Caragana Korshinskii and Pulp by BaLPMO10 and Cellulase

**Figure 6:**
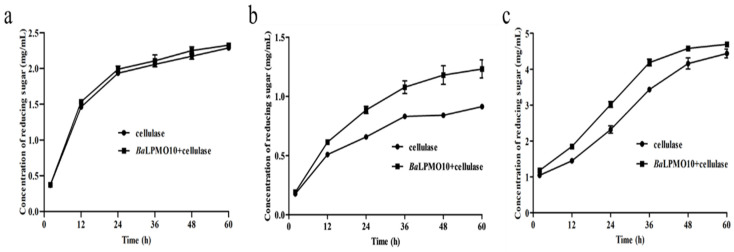
Cellulolytic activity of *Ba*LPMO10 on biomass. (**a**) Ethylenediamine-pretreated rice straw; (**b**) ethylenediamine-pretreated *Caragana korshinskii*; (**c**) NaOH-pretreated pulp.

Due to the complex composition of lignocellulosic substrates, different substrates were often chosen for the study of the activity of LPMO. To investigate the catalytic ability of *Ba*LPMO10 on various forms of plant biomass, experiments were conducted using rice straw treated with ethylenediamine, Caragana korshinskii treated with ethylenediamine and pulp treated with NaOH as substrates, which have frequently been used in previous studies [37,41,42,43]. The reducing sugar yield was increased after the synergistic degradation of all three substrates by *Ba*LPMO10 and cellulase compared to cellulase alone (Figure 6). Specifically, compared to the degradation of cellulose alone, the co-degradation of *Ba*LPMO10 and cellulase significantly increased the production of reducing sugars in Caragana korshinskii and pulp. However, the synergistic effect on rice straw was not significant, with only a 1.7–5.0% increase in reducing sugar production during the 2–60 h of synergistic degradation (Figure 6a). During the degradation of Caragana korshinskii, the production of reducing sugars gradually increased with the prolongation of the reaction time. Compared to cellulase alone, the efficiency of the synergistic degradation of the two enzymes reached a maximum at 48 h, with a 40.5% increase in reducing sugar production (Figure 6b). At 60 h, the efficiency decreased to 34.7%, possibly due to the gradual decrease in enzyme activity over time (Figure 6b). When degrading pulp, the best synergistic degradation effect was observed at 24 h, with a 30.1% increase in reducing sugar production compared to cellulase alone (Figure 6c). However, the synergistic degradation only increased the reducing sugar production by 5.7% at 60 h, with a concentration of 4.7 mg/mL (Figure 6c). At this point, the reducing sugar yield had already approached the initial substrate concentration of 5.0 mg/mL, indicating that most of the cellulose had been converted to reducing sugars. In summary, it could be seen that *Ba*LPMO10 demonstrated significant differences in conversion efficiency for these three biomasses, with the highest catalytic efficiency observed for Caragana korshinskii.

It can be seen that the degradation ability of *Ba*LPMO10 was different for various biomasses. This was similar to other LPMOs. For example, *Vi*LPMO10B synergized with cellulase on corn stalk, sugarcane bagasse and rice straw to increase the yields of reducing sugars by 17%, 16% and 22%, respectively, compared to cellulase alone [42]. Both *Mt*C1LPMO and its mutant R17L degraded rice straw more efficiently than corn straw and *Caragana korshinskii* [41]. The addition of PMO_07920 from *Aspergillus terreus* increased the conversion efficiency of oxalic acid-treated bagasse and rice straw by 4.68% and 17.10%, respectively [44]. LPMO9 from *Thermoascus aurantiacus* increased the conversion of organosolv-pretreated and steam-pretreated corn stover by 25% and 14%, respectively [15]. Additionally, the degradation capacity of *Ba*LPMO10 for biomass was higher than that of some LPMOs. For example, the synergistic action of LPMO with cellulase increased the conversion of the corn stover by 3–10% compared to cellulase alone [45]. Additionally, LPMO derived from *Chaetomium globosum* improved the conversion efficiency of filter paper by 5% [43].

Although commercial cellulase Celluclast 1.5 L has been widely used in the literature for the synergistic conversion of biomass with LPMO [10,13,37,43], the efficiency of the final reducing sugars produced in this study was still not high enough. As observed in previous studies [46], this may be due to the fact that Celluclast 1.5 L was not the most efficient enzyme for converting biomass. Therefore, it is also very important to optimize the enzyme preparation in future research. Moreover, the current reaction system produced low levels of reducing sugars, and unsuitable substrate concentrations may have also been a contributing factor. Currently, there is a trend towards hydrolysis at high concentrations of substrates [47]. Therefore, the conversion of suitable concentrations of biomass substrates into fermentable sugars using optimized enzyme preparations is well worthy of in-depth study.

**Figure 7:**
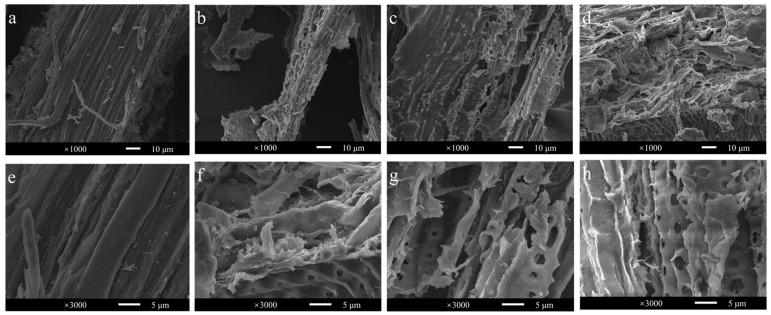
Observation of the morphology of ethylenediamine-pretreated *Caragana korshinskii* after enzymolysis. (**a**,**e**) Without enzyme; (**b**,**f**) with *Ba*LPMO10; (**c**,**g**) with cellulase; (**d**,**h**) both with *Ba*LPMO10 and cellulase. Figures (**a**–**d**) and (**e**–**h**) show the surface morphology of *Caragana korshinskii* at 1000× and 3000× magnification, respectively.

Since *Ba*LPMO10 had the highest degradation efficiency on *Caragana korshinskii*, to further analyze the structural changes of *Caragana korshinskii* during enzymatic digestion, the surface morphology of the substrate in four different reaction systems (without enzyme, *Ba*LPMO10 alone, cellulase alone, *Ba*LPMO10 and cellulase) was observed using SEM (Figure 7). The surface morphology of *Caragana korshinskii* at 1000× magnification is shown in Figure 7a–d. It can be seen that the surface of the unenzymatically degraded *Caragana korshinskii* was relatively flat (Figure 7a). After degradation of *Ba*LPMO10 (Figure 7b), the structure showed obvious porosity and fragmentation, indicating that *Ba*LPMO10 is able to disrupt the structure of *Caragana korshinskii*. After cellulase hydrolysis alone (Figure 7c), the surface of the *Caragana korshinskii* showed porosity, indicating that cellulase degraded it as well. The fracture and fragmentation of the structure of *Caragana korshinskii,* degraded synergistically by *Ba*LPMO10 and cellulase, were more pronounced (Figure 7d), indicating that the pretreatment of *Ba*LPMO10 increased the actionable zone of cellulase, thus making the structure of cellulose more susceptible to disruption.

To observe the surface morphology of *Caragana korshinskii* under different enzymatic conditions more clearly, observations were made at 3000× magnification, as shown in Figure 7e–h. The surface of untreated *Caragana korshinskii* was relatively smooth (Figure 7e), while the surface after *Ba*LPMO10 degradation was rough and porous (Figure 7f). In addition, the surface of the substrate was broken for both cellulase alone (Figure 7g) and *Ba*LPMO10 and cellulase synergistically (Figure 7h), but the substrate degraded by the two enzymes synergistically was more damaged, with a higher number of pores and larger pore sizes on the surface (Figure 7h). Many studies have reported that LPMOs can break the glycosidic bonds of polysaccharides [9,48], suggesting that the pretreatment with *Ba*LPMO10 broke the glycosidic bonds of cellulose and provided more action sites for other cellulase enzymes, promoting the efficient conversion of cellulose. Similar phenomena were also described in other studies. For example, microscopic observations revealed that *Gc*LPMO9s disrupted birch fibers [49]. The results of atomic force microscopy demonstrated the degradation of cellulose nanofibers into shorter and thinner insoluble fragments by LPMO [50,51]. SEM results showed that LPMO-R17L disrupted the structure of corn stover, making it broken and porous [37].

## 3. Materials and Methods

### 3.1. Optimization of IPTG Induction Concentration

The expression host used in this paper was the previously constructed *E. coli* BL21 (DE3) with the recombinant plasmid [25]. A single colony was selected and incubated in 5 mL of lysogeny broth (LB) containing 100 mg/L ampicillin at 37 °C and 220 rpm for 12 h to obtain the seed medium. Subsequently, 1 mL of the seed culture was transferred into 50 mL LB medium containing 100 mg/L ampicillin for fermentation. When the OD_600_ of the culture reached 0.6, IPTG was added at final concentrations of 0.1, 0.25, 0.5 and 1 mM, respectively. In addition, a final concentration of 1 mM CuSO_4_ was added to each medium, and the cultures were fermented at 16 °C for 20 h. After fermentation, the culture was centrifuged at 8000 rpm and 4 °C for 10 min to obtain the supernatant, which was then purified using Ni^+^-NTA affinity chromatography (Qiagen, Hilden, Germany) to obtain the target protein as previously described [25]. Protein concentrations were measured with a Nanodrop 2000 spectrophotometer (Thermo Fisher Scientific, Waltham, MA, USA) at 280 nm.

### 3.2. Optimization of Fermentation Temperature and Time

First, 1 mL of the seed culture from Section 2.1 was inoculated in 50 mL of LB medium containing 100 mg/L ampicillin. When the OD_600_ of the medium reached 0.6, IPTG and CuSO_4_ were added for final concentrations of 0.5 mM and 1 mM, respectively. The medium was then fermented at 16 °C, 30 °C and 37 °C for 4 h, 8 h and 20 h, respectively. After fermentation, the supernatant was separated from the medium by centrifugation (8000 rpm, 4 °C, 10 min) and purified using Ni^+^-NTA affinity chromatography (Qiagen, Hilden, Germany) to obtain the target protein, as previously described [25]. The protein purity was analyzed by SDS-PAGE.

### 3.3. SDS-PAGE Analysis

A 20 μL sample was mixed with 5 μL loading buffer, boiled for 10 min and centrifuged at 5000 rpm for 2 min. Then, 10–15 μL of the supernatant was loaded onto SDS-PAGE gels (5% for stacking gels and 12% for separating gels), and gel electrophoresis was performed under constant pressure. After electrophoresis, the gels were placed in Coomassie Brilliant Blue, microwaved for 30 s to fix the color and stained with slight shaking at room temperature for 1 h. After removal of the Coomassie Brilliant Blue, water was added to cover the stained gels and heated for about 10 min for decolorization until the protein bands were clear.

### 3.4. Enzyme Activity Assay

The enzymatic activity of *Ba*LPMO10 was determined using 2,6-dimethoxyphenol (2,6-DMP) as substrate and H_2_O_2_ as co-substrate [32]. The 250 μL reaction system included 195 μL of 50 mM pH 6.0 sodium phosphate buffer, 25 μL of 10 mM 2,6-DMP solution, 5 μL of 50 mM H_2_O_2_ stock solution and 25 μL of purified *Ba*LPMO10 at a concentration of 0.5 mg/mL. The enzyme activity was calculated by measuring the absorbance value after 5 min of reaction at 469 nm using multimode reader. One unit of enzyme activity is defined as the amount of enzyme required to generate 1 µmol of oxidation product (ε_469_ = 53,200 M^−1^ cm^−1^) per minute under reaction conditions.

### 3.5. Effect of Metal Ions, DTT, EDTA and Organic Reagents on the Enzyme Activity of BaLPMO10

Metal ions (K^+^, Na^+^, Ca^2+^, Ba^2+^, Mg^2+^, Cu^2+^, Mn^2+^, Co^2+^ and Fe^3+^), reducing agent DTT and chelating agent EDTA were added to the reaction system at final concentrations of 1 mM, 5 mM and 10 mM, respectively, and the enzyme activity of *Ba*LPMO10 was determined at the optimum temperature and pH. Methanol, ethanol, ethylene glycol, polyethylene glycol and isopropyl alcohol were added to the reaction system at 10%, and the residual activity of *Ba*LPMO10 was determined under optimum reaction conditions. The enzyme activity of *Ba*LPMO10 without the above added substances was taken as 100%. All experiments were repeated three times.

### 3.6. Determination of Enzymatic Kinetics of BaLPMO10

The *K*_m_ and *V*_max_ values of *Ba*LPMO10 were determined through reactions with different concentrations of 2,6-DMP (1–50 mM) at 45 °C for 5 min in 50 mM sodium phosphate buffer (pH 6.0). All reactions were performed in triplicate. The curves and kinetic parameters were analyzed using GraphPad Prism software.

### 3.7. Enzymatic Degradation of Different Biomasses by BaLPMO10

To evaluate the degradation activity of *Ba*LPMO10 on different pretreated biomasses, two groups of experiments were conducted. One group utilized corn stover pretreated by steam explosion at 200 °C for 3, 6, 9 and 12 min, while the other group used three types of biomasses: rice stover pretreated with ethylenediamine, Caragana korshinskii pretreated with ethylenediamine and bleached softwood kraft pulp pretreated with dilute NaOH. The reaction system consisted of 1 mL of 50 mM pH 6.0 sodium phosphate buffer, including 5 mg substrate, 20 μL 1 mg/mL *Ba*LPMO10 and 10 μL 100 mM ascorbic acid. A reaction system without LPMO was used as a blank control. The system was reacted for 24 h at 30 °C and 900 rpm on a shaker, followed by the addition of 1 μL of commercial cellulase Celluclast 1.5 L (Novozymes, Bagsvaerd, Denmark), and the reaction was continued for 2–60 h. The reaction was terminated by boiling for 10 min. The supernatant was obtained by centrifugation at 12,000 rpm for 10 min, and the concentration of reducing sugar was measured using dinitrosalicylic acid (DNS) reagent [52]. A 100 μL sample of the supernatant was added to a 1.5-mL Ep tube and mixed with 300 μL of DNS. The mixture was reacted accurately in boiling water for 5 min, and then the reaction was terminated in an ice bath for 2 min. After centrifugation at 5000 rpm for 2 min, 200 μL was taken into a 96-well plate, and the absorbance value at OD_540_ nm was measured using a multimode reader to calculate the reducing sugar content.

### 3.8. Surface Morphology Analysis

The surface morphology of the biomass was examined using a scanning electron microscopy (SEM). The samples to be observed were freeze-dried and fixed on a sample stage using conductive adhesive. To remove the surface charge, a layer of gold particles was sprayed onto the sample surface, and then the surface morphology was observed with a SU1510 scanning electron microscope under an accelerating voltage of 5 kV.

## 4. Conclusions

In this study, the bioinformatics of *Ba*LPMO10 were analyzed, and it was found to be a secreted protein. Its highest secretion level was induced by 0.5 mM IPTG and fermented at 37 °C for 20 h, and 20 mg of protein with >95% purity was obtained by purifying 1 L of supernatant. It was found that 10 mM of Ca^2+^ and Na^+^ increased the enzyme activity of *Ba*LPMO10 by 47.8% and 98.0%, respectively. However, DTT, EDTA and five organic reagents inhibited the enzyme activity of *Ba*LPMO10. *Ba*LPMO10 had the highest synergistic degradation efficiency with cellulase on steam-exploded corn stover at 200 °C for 12 min, increasing the reducing sugars by 9.2% compared to cellulase alone. The degradation of three different biomasses revealed that *Ba*LPMO10 had the highest activity against ethylenediamine-pretreated Caragana korshinskii, and the reducing sugar content increased by 40.5% after 48 h of degradation by *Ba*LPMO10 in synergy with cellulase compared to cellulase alone. The observation of the surface morphology revealed that *Ba*LPMO10 was able to disrupt the cellulose structure and increase the accessibility of cellulase, thus facilitating the conversion process. Considering the importance of LPMO in the conversion of lignocellulose, this study provides theoretical support for achieving efficient conversion of plant biomass.

## Figures and Tables

**Table 1:** Kinetic parameters of *Ba*LPMO10.

Substrate	*V*_max_ (U/g)	*K*_m_ (mmol/L)	*k*_cat_ (s^−1^)	*k*_cat_/*K*_m_ (s^−1^ mM^−1^)
2,6-DMP	21.78 ± 2.23	5.89 ± 1.95	108.9 ± 11.15	18.48

## Data Availability

The data analyzed in this study are included in the paper.

## Supplementary Materials

The following supporting information can be downloaded at: https://www.mdpi.com/article/10.3390/ijms24119710/s1.
