# Supplementary material for: Efficient Secretory Production of Lytic Polysaccharide Monooxygenase BaLPMO10 and Its Application in Plant Biomass Conversion
Source: Int J Mol Sci. 2023 Jun 3;24(11):9710. doi: 10.3390/ijms24119710 (PMC10253640; doi:10.3390/ijms24119710)
Supplement: Supplementary file 1 [file ijms-24-09710-s001.zip › ijms-2371381-supplementary.pdf]

Figure S1.

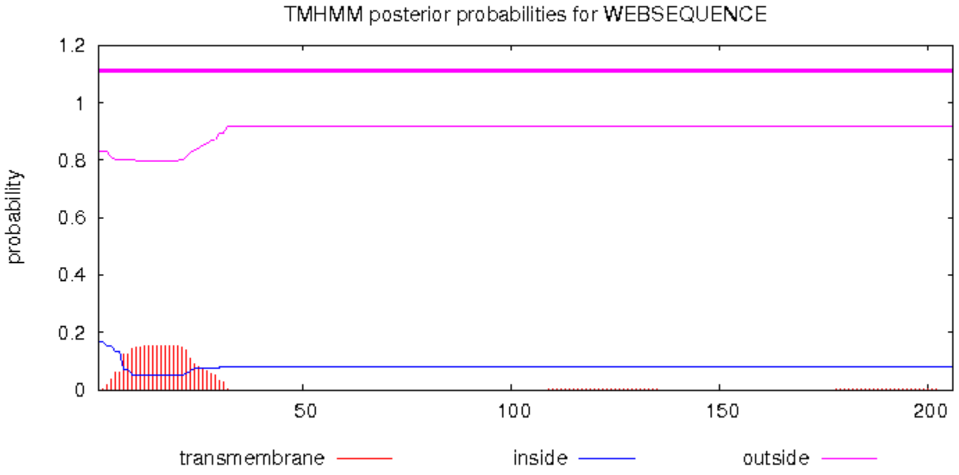

Figure S1 Prediction of transmembrane domain in *Ba*LPMO10

Figure S2.

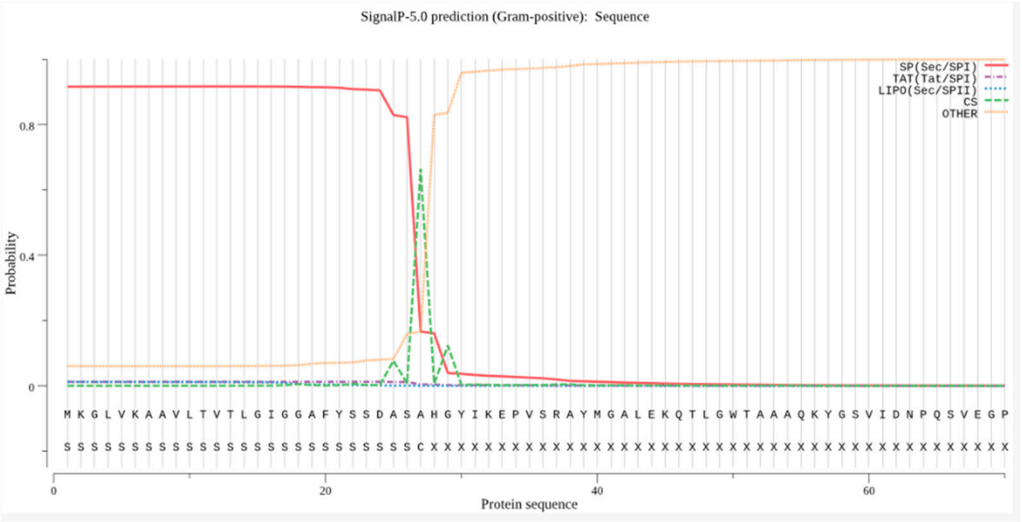

Figure S2 Signal peptide prediction of *Ba*LPMO10
